# Demographic survey of pediatric patients presenting to a chiropractic teaching clinic

**DOI:** 10.1186/1746-1340-18-33

**Published:** 2010-12-15

**Authors:** Joyce Miller

**Affiliations:** 1 Anglo-European College of Chiropractic, 13-15 Parkwood Road, Bournemouth, UK

## Abstract

**Background:**

Considering the increasing use of alternative therapies for children, it is appropriate to determine the demographic profile of pediatric patients entering a chiropractic clinic.

**Methods:**

Collection of demographic data including age, gender, condition at presentation, previous clinicians consulted and medical referral rates of pediatric patients presenting to a chiropractic teaching clinic between 2006 and 2010.

**Results:**

Over-all, 20.5% of patients were aged between two days and 15 years and classified as pediatric patients. The most common presenting complaint was musculoskeletal (35%). Excess crying (30%) was the most common complaint in the largest presenting age group which was under 12 weeks of age (62.3%). All children had previously presented for medical care for the same condition. Most (83%) of the infant patients under 12 weeks of age were referred for care by a medical practitioner.

**Conclusion:**

Parents commonly presented their child for care at this chiropractic clinic with a recommendation from a medical practitioner. The most common complaints were musculoskeletal and excessive crying conditions and the most prevalent age group was under 12 weeks of age.

## Introduction

The use of complementary and alternative medicine (CAM) by the pediatric population is increasing [1]. A recent study by Vlieger et al found that perceived adverse effects of allopathic medication, low effect of conventional treatment, school absenteeism and age less than 11 years were predictors of use of CAM care for children in the Netherlands [2]. It is estimated that 11.8% of children in the USA use CAM therapies [3]. The Center for Disease Control in the USA reported that manual therapy was the most common type of practitioner-based CAM therapy chosen for children and that musculoskeletal conditions were the most common types of conditions for which treatment was sought [4]. A 2007 Canadian study corroborated these findings, stating that musculoskeletal care was the most common type of CAM treatment chosen by parents for their children. Personal experience, lack of appropriate treatments available from conventional medicine or referral from a physician were the reasons given for seeking alternative care [5].

The aim of this study was to investigate the pediatric patients who attended a university-affiliated chiropractic teaching clinic on the south coast of England between 2006 and 2010. The goals were to determine the frequency of presentation in each age group, reasons for attendance, referral patterns and usage of other types of health care prior to presentation and demographic features.

## Methods

The data presented in this report were obtained from a computerised system maintained by clinicians overseeing the care of pediatric patients up to 16 years of age between January 2006 and January 2010. This was a cross-sectional study of baseline demographic data of pediatric patients presenting to the Anglo-European College of Chiropractic (AECC) outpatient clinic.

Descriptive statistical analysis was performed using Microsoft Excel. All data were held confidentially. Parents consented that the data could be used for research purposes. Ethical approval was granted by the AECC Projects Panel.

Complaints were categorised as musculoskeletal if they pertained to the axial or appendicular skeleton or resulted in a dysfunction or discomfort of movement or posture.

## Results

**Figure 1:**
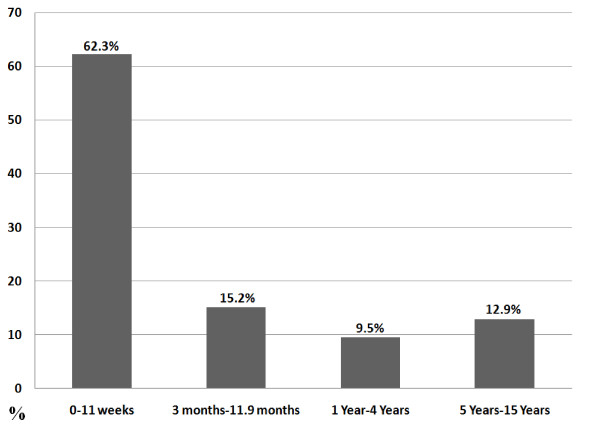
A**ges of pediatric patients presenting to Anglo-European College of Chiropractic 2006-2010; N = 2645**.

**Figure 2:**
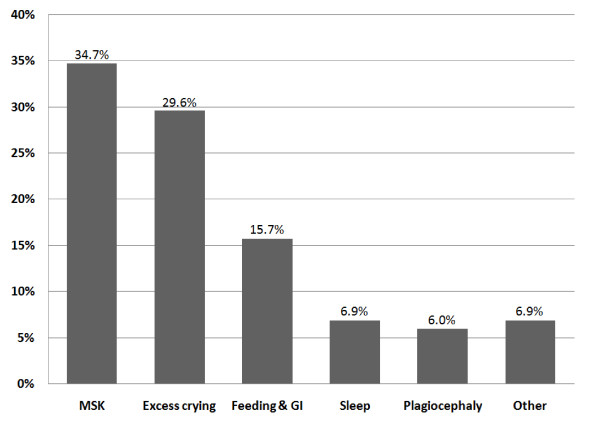
**Conditions for which pediatric patients presented to Anglo-European College of Chiropractic 2006-2010; N = 2645**. Abbreviations: MSK = musculoskeletal, GI = gastrointestinal.

**Figure 3:**
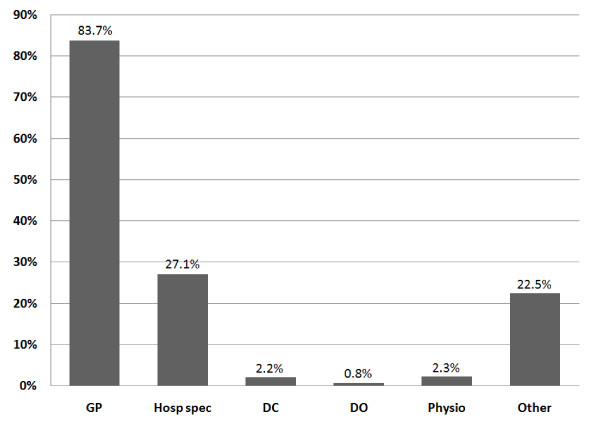
**Health care providers visited for same condition prior to pediatric presentation to Anglo-European College of Chiropractic; N = 2645**. Abbreviations: GP = general practitioner; Hosp Spec = hospital specialist; DC = doctor of chiropractic; DO = doctor of osteopathy; Physio = physiotherapist.

**Figure 4:**
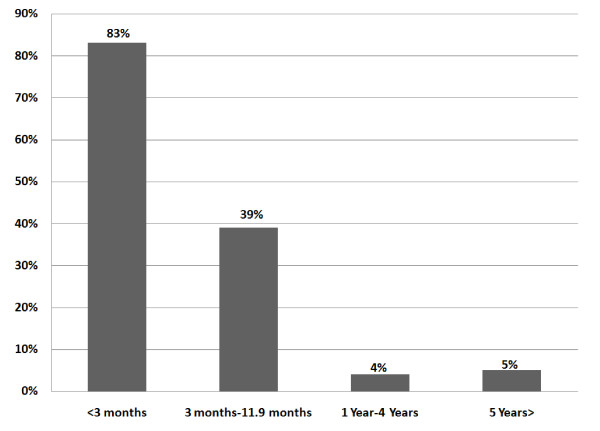
**Medical rates of referral of pediatric patients presented to Anglo-European College of Chiropractic teaching clinic 2006-2010; N = 2645**. Abbreviations: < = less than; > = greater than.

This data system included 2,645 pediatric patients (0-15 years of age). Of these, 2,303 (87%) were under the age of five and 342 (13%) were between the ages of 5 and 15 (Figure 1). Fifty-seven percent were male and 43% were female. These patients were categorised according to complaint on presentation (Figure 2). The complaints of all children over five years were categorized as musculoskeletal. In all age groups, just over a third (34.7%) presented with musculoskeletal problems, 29.6% presented with excess crying (previously known as infant colic) and 15.7% with feeding disorders. All children had previously presented to at least one medical practitioner for the same condition and some had presented to multiple healthcare practitioners (Figure 3). The younger the child, the more common the referral with 83% of infants under 12 weeks of age being sent by a medical practitioner, 39% between 3 and 12 months of age and a 4-5% rate of referral in age groups over one year. Figure 4 shows referral rates relative to age group. Over-all, 20.5% of the clinic patients were aged between two days and 16 years.

## Discussion

Boys were more commonly presented than girls. This may be due to the prevalence of musculoskeletal health problems which have previously been shown to be more common in boys [6]. The patient proportions in that study (57.4% male) versus girls (42.6%) [6] were virtually the same as in our clinic (57% male and 43% female). At birth, boys are often larger than girls and intra-uterine constraint may result in biomechanical imbalance or asymmetries in their cranium, spine or extremities [7].

It is not surprising that musculoskeletal problems were the most common presentation of the pediatric patient to our clinic. First, chiropractors are known to specialize in the musculoskeletal system and second, musculoskeletal pain affects a significant number of children [6]. Further, these conditions carry a significant economic burden due to time lost at school, lost time from work for parents and diagnostic procedures and referrals and consultation with multiple practitioners [6]. What may be surprising is that parents have heightened awareness to recognize musculoskeletal pain in the very youngest children, particularly neonates. A common complaint of early infancy is that the baby "refuses" to lie on his/her back (shows pain behaviours when lying supine) [8]. The "back to sleep" program is required for cot death prevention [9]. Manual therapy might be considered useful to treat the infant to help the infant to sleep comfortably [10]. Manual therapy was the most commonly chosen therapy by parents for their child in a USA study [4]. In a recent UK survey, clinicians (pediatricians, orthopedists, primary care (both new trainees and experienced) and emergency medicine doctors were asked how confident they felt dealing with pediatric musculoskeletal (pMSK) problems. Seventy-four percent had "no" or "some confidence" [11]. It is possible that clinicians with little confidence to treat pMSK problems may refer these cases to manual therapists. In a London study of general practitioners, 83% had referred for CAM therapies or influenced such referral [12], although this study was not specific to pMSK.

Referrals to this chiropractic teaching clinic from medical professionals were common. Children under three months of age had the highest (83%) referral rates. It is not surprising that medical professionals referred pediatric patients to this clinic for musculoskeletal conditions such as torticollis and other postural preferences that cause difficulty and perhaps even pain when the infant is moved out of their antalgic posture. However, crying and feeding problems were also commonly referred. These early "quality of life" problems such as excess crying (previously known as infant colic) and feeding problems as well as sleep dysomnias may be considered to be amenable to biomechanical attention [13]. However, the efficacy of chiropractic care for these conditions has not yet been proven or disproven [14]. There are some suggestions that feeding problems in the neonate may be biomechanical in nature [15] and one study suggests that multidisciplinary care which included chiropractic may be helpful [16]. There may also be benefit to ruling out a simple musculoskeletal lesion which could be corrected quickly with little risk before the child undergoes more invasive testing or procedures.

The population most commonly presented by their parents for care in this study were young, under 12 weeks of age. These results are similar to a Danish study that found the most common pediatric patients to present to chiropractors were under four months of age [17].

## Conclusion

In this chiropractic clinic, pediatric patients most commonly presented for excessive crying in the early months and for musculoskeletal complaints at all ages. Parents often brought their child to this clinic on the recommendation of medical professionals, particularly in the infant population. All children had consulted a medical practitioner prior to their presentation to this clinic. Further research is required to ascertain therapeutic benefit, cost/benefit and rates of satisfaction for this type of treatment.

## Competing interests

The authors declare that they have no competing interests.
